# Ultra-early computed tomography markers of haematoma expansion: Potential trial targets?

**DOI:** 10.1093/esj/23969873251355938

**Published:** 2026-01-01

**Authors:** Chloe A Mutimer, Sameer Sharma, Henry Zhao, Atte Meretoja, Leonid Churilov, Teddy Y Wu, Timothy J Kleinig, Philip M Choi, Andrew Cheung, Jiann-Shing Jeng, Henry Ma, Duy Ton Mai, Huy-Thang Nguyen, Gagan Sharma, Bruce C V Campbell, Geoffrey A Donnan, Stephen M Davis, Nawaf Yassi

**Affiliations:** Department of Medicine and Neurology, Melbourne Brain Centre, The Royal Melbourne Hospital, University of Melbourne, Parkville, Australia; Department of Medicine and Neurology, Melbourne Brain Centre, The Royal Melbourne Hospital, University of Melbourne, Parkville, Australia; Department of Neurology, Alfred Hospital, Melbourne, VIC, Australia; Department of Medicine and Neurology, Melbourne Brain Centre, The Royal Melbourne Hospital, University of Melbourne, Parkville, Australia; Department of Neurology, Helsinki University Hospital, Helsinki, Finland; Department of Medicine and Neurology, Melbourne Brain Centre, The Royal Melbourne Hospital, University of Melbourne, Parkville, Australia; Melbourne Medical School, University of Melbourne, Parkville, VIC, Australia; Department of Neurology, Christchurch Hospital, Christchurch, New Zealand; Department of Medicine, University of Otago, Christchurch, New Zealand; Department of Neurology, Royal Adelaide Hospital, Adelaide, SA, Australia; Department of Neuroscience, Box Hill Hospital, Eastern Health, Eastern Health Clinical School, Monash University, Box Hill, VIC, Australia; Department of Interventional Neuroradiology, Liverpool Hospital, Liverpool, NSW, Australia; Department of Neurology, National Taiwan University Hospital, Taipei, Taiwan; Department of Medicine, School of Clinical Sciences, Monash University, Melbourne, VIC, Australia; Stroke Centre, Bach Mai Hospital, Hanoi Medical University, VNU University of Medicine and Pharmacy, Hanoi, Viet Nam; Department of Cerebrovascular Disease, 115 Hospital, Ho Chi Minh City, Viet Nam; Department of Medicine and Neurology, Melbourne Brain Centre, The Royal Melbourne Hospital, University of Melbourne, Parkville, Australia; Department of Medicine and Neurology, Melbourne Brain Centre, The Royal Melbourne Hospital, University of Melbourne, Parkville, Australia; Department of Medicine and Neurology, Melbourne Brain Centre, The Royal Melbourne Hospital, University of Melbourne, Parkville, Australia; Department of Medicine and Neurology, Melbourne Brain Centre, The Royal Melbourne Hospital, University of Melbourne, Parkville, Australia; Department of Medicine and Neurology, Melbourne Brain Centre, The Royal Melbourne Hospital, University of Melbourne, Parkville, Australia; Population Health and Immunity Division, The Walter and Eliza Hall Institute of Medical Research, Parkville, VIC, Australia

**Keywords:** Intracerebral haemorrhage, computed tomography, prognosis, haematoma expansion

## Abstract

**Introduction:**

The predictive value of CT markers of intracerebral haemorrhage (ICH) expansion is time-dependent, but data in the ultra-early period (<2 h from onset) are limited. We aimed to describe the frequency of these CT markers, their association with haematoma volume, haematoma expansion (HE) and functional outcome at 90-days. We also investigated the effect of tranexamic acid on HE in the presence of these markers.

**Patients and methods:**

We performed a pooled analysis of individual patient data from the STOP-AUST and STOP-MSU placebo-controlled randomised trials of tranexamic acid, including ICH patients scanned within 2 h of symptom onset. Logistic regression was used to assess the association between CT markers and HE or 90-days functional outcomes (poor outcome defined as mRS3-6).

**Results:**

Among 246 patients, the swirl sign (74.3%) was the most frequent CT marker and the blend sign least frequent (7.3%). All markers were associated with increased baseline haematoma volume, and excluding the black hole sign, all were more common in patients with 24-h HE. The blend and spot signs were associated with 24-h HE and heterogenous density, swirl sign, hypodensity and island sign were associated with poor 90-day function outcomes in univariate logistic regression. However, the area under the receiver-operating-characteristic curve was similar for all markers and indicated low discriminative ability (Chi-squared test *p* = 0.81). A potential benefit of tranexamic acid in HE reduction was observed in patients with the spot sign (interaction *p* = 0.01)

**Conclusions:**

The discriminative utility of CT markers of HE in the early timeframe appears insufficient. There may be an effect of tranexamic acid in spot sign positive patients <2 h from onset.

## Graphical Abstract

**Graphical abstract:**
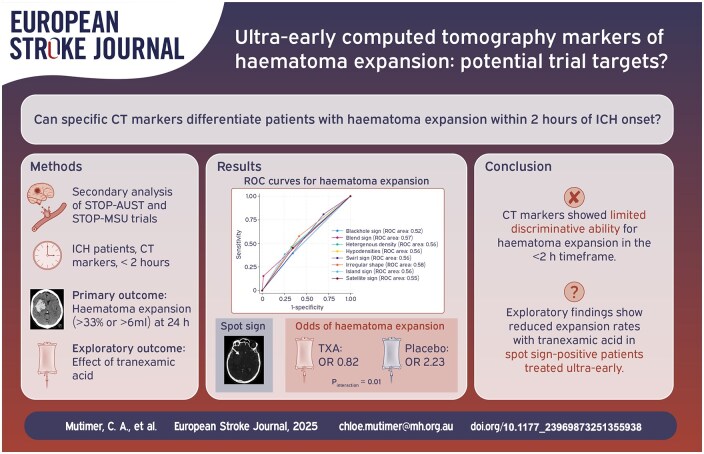

## Introduction

Haematoma expansion in primary intracerebral haemorrhage is identifiable in up to one-third of patients within the first 24 h, mostly in the first few hours and is strongly associated with neurological deterioration, poor functional outcome and mortality.^1,2^ A number of clinical trials in intracerebral haemorrhage have attempted to reduce haematoma expansion, however, reliably identifying a target population has been challenging.^3–9^

CT markers of haematoma expansion have been described in the literature, with the non-contrast CT markers (NCCT) broadly classified as shape and density markers.^10,11^ NCCT markers are attractive candidate biomarkers for selection into trials as they use routinely acquired imaging without the need for intravenous contrast, and minimal image post-processing. In addition, quantitative assessment of mean haematoma density (mean Hounsfield units within the haematoma) has also been reported on NCCT, with lower values associated with haematoma expansion.^12^ Time from symptom onset to CT is known to affect the predictive value of these markers, with limited data in the ultra-early time period.^13^

The spot sign, representing contrast extravasation on CT-angiography within the haematoma, is a surrogate marker of ongoing bleeding, and is associated with haematoma expansion, particularly in the early time period.^14^ The spot sign has been used to select patients for trials of haemostatic therapy with neutral results to date.^3,15^

To better understand the association of these CT markers with haematoma expansion in the ultra-early timeframe, we performed a pooled descriptive analysis of the STOP-AUST and STOP-MSU randomised controlled trials.^3,4^ We aimed to (a) describe the frequency of CT markers of haematoma expansion in the ultra-early period of ICH, (b) describe their association with baseline haematoma volume, 24-h haematoma expansion and functional outcomes, (c) evaluate their discriminative performance for haematoma expansion and (d) investigate the effect of tranexamic acid on haematoma expansion in the presence of these markers.

## Methods

### Study design and participants

Participants were from the STOP-AUST (2013–2019)^3^ and STOP-MSU (2018–2023)^4^ international multicentre randomised controlled trials of primary intracerebral haemorrhage. STOP-AUST compared intravenous tranexamic acid to placebo in patients with a CT-angiography spot sign within 4.5 h of symptom onset and STOP-MSU compared the same intervention in patients presenting within 2 h of symptom onset and did not require presence of a CT-angiography spot sign. The full inclusion and exclusion criteria for each trial is available in the Supplemental Material. Only patients with baseline imaging within 2 h of symptom onset with available baseline and 24-h imaging were included in the present study. The manuscript was prepared following STROBE guidelines.

### Standard protocol approvals, registrations and patient consents

The STOP-AUST (NCT01702636) and STOP-MSU (NCT03385928) study protocols were approved by the respective ethics committees of the recruiting sites. Written informed consent was obtained from the participant or a legal representative before enrolment according to the Declaration of Helsinki. Emergency treatment followed by consent for continued participation was allowed in some jurisdictions.

### Neuroimaging analysis

**Figure 1.**
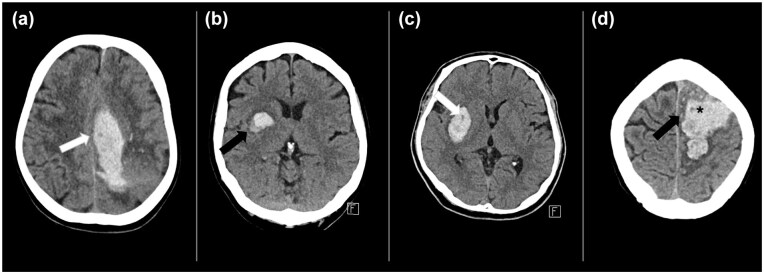
examples of non-contrast CT markers of haematoma expansion. (a) Lobar haematoma with concurrent signs of heterogenous density, swirl sign and hypodensities (overlapping signs, all indicated by white arrow). (b) Deep haematoma with blend sign (black arrow). (c) Deep haematoma with black hole sign (white arrow). (d) Lobar haematoma with irregular shape and satellite sign (black arrow). This haematoma also has heterogenous density, hypodensities and swirl signs (overlapping signs, both indicated by the star).

Non-contrast CT markers were defined as per a 2019 review article^10^ with examples shown in Figure 1. These markers include the density signs (black hole sign, blend sign, heterogenous density, hypodensities, swirl sign) and the shape signs (irregular shape, island sign, satellite sign). All imaging markers were reviewed separately by two authors (CM and SS, neurologists), with discrepancies resolved by agreement; both raters were blinded to the outcomes of interest. The spot sign was adjudicated by the same two authors for the STOP-MSU patients, and by the central imaging lab from the STOP-AUST trial (two readers after database lock). The spot sign definition (Appendix) used was the same for both datasets.^3^ Each CT marker was assessed independently in this analysis, and co-existence of multiple CT markers within the same patient was permitted. Haematoma volumes (intracerebral and intraventricular) was measured using non-contrast CT and a validated semi-automated planimetric method,^16^ with baseline and 24-h imaging assessed as part of the original trial by a central imaging lab. Quantitative markers of density, mean haematoma Hounsfield units (HU) calculated by the semi-automated programme using a threshold range of 40–100 HU, the standard deviation and maximum HU were also obtained (Supplemental Figure S2). This measurement was only reported for patients scanned in-hospital (i.e. not on a mobile stroke unit CT scanner). The standard deviation of the haematoma HU is referred to as HU variability. The HU coefficient of variation was calculated by dividing the standard deviation by the mean.

#### Outcomes

Haematoma expansion at 24-h compared to baseline was defined as an absolute increase of ⩾6 ml or a relative increase of ⩾33% in parenchymal haematoma volume (as defined in the original trials).^17^ Poor functional outcome at 90-days was defined as mRS 3–6.

#### Statistical analysis

We compared baseline clinical features, radiological and clinical outcomes of patients using Fisher’s exact test for categorical outcomes and Mann-Whitney U test for continuous outcomes as appropriate. This was performed for patients: (a) with and without haematoma expansion and (b) with and without each CT marker. To investigate agreement between reviewers for each non-contrast CT marker, Cohen’s kappa test was performed.

We then tested whether each CT marker was associated with haematoma expansion and functional outcome in univariable logistic regression. Given the descriptive nature of this study, no adjustment for confounding was performed for the primary analysis.^18–20^ Sensitivity analysis for the same outcomes was performed using multivariable logistic regression, adjusting for age and baseline haematoma volume, in keeping with previous studies.^21–24^ Given that eight CT markers were tested for association with haematoma expansion, we applied a Bonferroni correction for multiple comparisons, setting the adjusted significance threshold at *p* < 0.00625 (0.05/8). We only assessed this on our primary outcome. Spearman correlation was used to describe the association between heterogenous density and quantitative Hounsfield unit data. The discriminative performance of each CT marker was evaluated for haematoma expansion using the area under the curve, sensitivity, specificity, positive predictive value and negative predictive value. Differences in area under the receiver-operator-curve between the definitions were assessed using a Chi-squared test.

Finally, to investigate whether the association between treatment group (tranexamic acid or placebo) and haematoma expansion was modified by the presence of a CT marker, we included a multiplicative interaction term (CT marker × treatment group) in a logistic regression model.

Statistical analyses were performed with STATA-SE version 18 (StataCorp. College Station, TX), and two-sided *p-*values ⩽ 0.05 were considered statistically significant.

## Results

Of the 301 patients enrolled in STOP-AUST and STOP-MSU, there were 246 patients with both baseline and 24-h imaging available for analysis and 212 with CT angiography available (Supplemental Figure 1).

Baseline characteristics of the patients are shown in Table 1. There were 105 patients (42.7%) with haematoma expansion at 24 h. Patients with haematoma expansion were older, had larger baseline intracerebral haemorrhage volumes, with a higher baseline NIHSS and lower baseline GCS indicating more severe clinical syndromes.

**Table 1.** baseline and follow-up clinical and imaging characteristics.

Characteristic	Total patients (*n* = 246)	24-h haematoma expansion (*n* = 105)	No 24-h haematoma expansion (*n* = 141)	*p*-Value
*Demographics*				
Age, median (IQR)	67 (57–77)	71 (59–79)	66 (54–75)	0.006
Male sex, *n* (%)	148 (60.2%)	58 (55.2%)	90 (63.9%)	0.173
Region				0.388
Australia/New Zealand, *n* (%)	171 (69.5%)	78 (74.3%)	93 (69.5%)	
Asia, *n* (%)	45 (18.3%)	16 (15.2%)	29 (20.6%)	
Finland, *n* (%)	30 (12.2%)	11 (10.5%)	19 (13.5%)	
Baseline mRS, median (IQR)	0 (0–0)	0 (0–0)	0 (0–0)	0.995
Past medical history				
Diabetes mellitus, *n* (%)	40 (16.3%)	16 (15.2%)	24 (17.0%)	0.708
Atrial fibrillation, *n* (%)	12 (4.9%)	8 (7.6%)	4 (2.8%)	0.085
Prior ischaemic stroke or TIA, *n* (%)	25 (10.2%)	12 (11.4%)	13 (9.2%)	0.571
Prior ICH, *n* (%)	13 (5.3%)	6 (5.7%)	7 (5.0%)	0.795
Antiplatelet use, *n* (%)	58 (23.6%)	31 (29.5%)	27 (19.1%)	0.058
*Clinical features*				
Baseline SBP (mmHg), median (IQR)	166 (150–183)	166 (150–182)	165 (151–184)	0.447
Baseline DBP (mmHg), median (IQR)	90 (79–104)	90 (78–104)	90.5 (80–103)	0.331
Baseline NIHSS, median (IQR)	13 (9–19)	16 (10–20)	12 (8–17)	0.001
Baseline GCS, median (IQR)	15 (13–15)	14 (12–15)	15 (14–15)	0.046
Onset to scan time (mins), median (IQR)	75 (60–88)	76 (57–91)	75 (61–87)	0.634
Randomised to tranexamic acid, *n* (%)	124 (50.4%)	51 (48.6%)	73 (51.8%)	0.619
*Baseline imaging features*				
ICH location				0.028
Lobar, *n* (%)	48 (19.5%)	27 (25.7%)	21 (14.9%)	
Deep, *n* (%)	197 (80.1%)	77 (73.3%)	120 (85.1%)	
Cerebellar, *n* (%)	1 (0.4%)	1 (1.0%)	0 (0%)	
Presence of IVH, *n* (%)	58 (23.6%)	16 (15.2%)	42 (30.0%)	0.007
Baseline ICH volume (ml), median (IQR)	10.8 (5.8–21.9)	15.5 (7.8–28.4)	8.4 (4.1–16.4)	<0.001
Baseline IVH volume (ml), median (IQR)	0 (0–0)	0 (0–0)	0 (0–0.5)	0.031
*24-h imaging features*				
ICH volume (ml), median (IQR)	15.2 (7.2–35.0)	33.3 (15.6–67.9)	8.7 (4.5–16.7)	<0.001
IVH volume (ml), median (IQR)	0 (0–3.3)	0 (0–4.1)	0 (0–2.8)	0.095
Absolute ICH growth (ml), median (IQR)	1.8 (−0.04 to 9.4)	13.3 (6.2–27.0)	0.1 (−0.5 to 1.0)	<0.001
Percentage ICH growth (%), median (IQR)	17.8 (−1.0 to 61.3)	76.5 (47.3–146.2)	3.0 (−5.3 to 11.9)	<0.001
*Outcomes*				
90-day mRS, median (IQR)	3 (2–5)	4 (3–6)	3 (2–4)	<0.001
90-day poor outcome, *n* (%)	176 (71.5%)	90 (85.7%)	86 (61.0%)	<0.001
90-day mortality, *n* (%)	42 (17.1%)	32 (30.5%)	10 (7.1%)	<0.001

The kappa score was >0.80 for all CT markers between two reviewers, indicating excellent interrater reliability (Supplemental Table S1). Frequency of markers is shown in Table 2. Only one patient had a fluid level sign, and given the rarity in our participant cohort, further analysis for this sign was not performed.

**Table 2.** Frequency of CT markers and association of treatment with haematoma expansion.

CT marker present	Number	Frequency	24-h haematoma expansion	No 24-h haematoma expansion	OR (95%CI)	*p*-Interaction
Heterogenous density (*n* = 246)						
Present	95	38.6%	48 (50.5%)	47 (49.5%)		
Tranexamic acid	43		18 (41.9%)	25 (58.1%)	0.72 (0.39–1.32)	*p* = 0.09
Placebo	52		30 (57.7%)	22 (42.3%)	1.36 (0.79–2.36)	
Hypodensities/swirl sign (*n* = 246)						
Present	183	74.3%	85 (46.4%)	98 (53.6%)		
Tranexamic acid	93		42 (45.2%)	51 (54.8%)	0.82 (0.55–1.24)	*p* = 0.82
Placebo	90		43 (47.8%)	47 (52.2%)	0.91 (0.60–1.38)	
Black hole sign (*n* = 246)						
Present	90	36.3%	41 (45.6%)	49 (54.4%)		
Tranexamic acid	49		18 (36.7%)	31 (63.3%)	0.58 (0.32–1.04)	*p* = 0.06
Placebo	41		23 (56.1%)	18 (43.9%)	1.28 (0.69–2.37)	
Blend sign (*n* = 246)						
Present	18	7.3%	16 (88.9%)	2 (11.1%)		
Tranexamic acid	9		8 (88.9%)	1 (11.1%)	8.00 (1.00–63.9)	*p* = 0.93
Placebo	9		8 (88.9%)	1 (11.1%)	8.00 (1.00–63.9)	
Irregular shape (*n* = 246)						
Present	119	48.6%	60 (50.4%)	59 (49.6%)		
Tranexamic acid	58		29 (50%)	29 (50%)	1.00 (0.60–1.67)	*p* = 0.70
Placebo	61		31 (50.8%)	30 (39.2%)	1.03 (0.63–1.71)	
Island sign (*n* = 246)						
Present	76	30.9%	40 (52.6%)	36 (47.4%)		
Tranexamic acid	39		20 (51.3%)	19 (52.8%)	1.11 (0.71–1.74)	*p* = 0.55
Placebo	37		20 (54.1%)	17 (45.9%)	1.18 (0.62–2.25)	
Satellite sign (*n* = 246)						
Present	96	39%	47 (49.0%)	49 (51.0%)		
Tranexamic acid	49		20 (40.8%)	29 (59.2%)	0.69 (0.39–1.22)	*p* = 0.09
Placebo	47		27 (57.5%)	20 (52.5%)	1.35 (0.76–2.41)	
Spot sign (*n* = 212)						
Present	84	39.6%	48 (57.1%)	36 (42.9%)		
Tranexamic acid	42		19 (45.2%)	23 (54.8%)	0.83 (0.45–1.52)	*p* = 0.01
Placebo	42		29 (69%)	13 (31%)	2.23 (1.16–4.29)	

There were 174 patients (68.2%) with appropriate imaging for quantitative density analysis (mobile stroke unit enrolment *n* = 44, without other appropriate baseline imaging *n* = 37). The mean haematoma density was 59.8 ± 4.5 HU (range 50.5–81.3), HU variability was 9.3 ± 1.3 (range 6.6–13.4) and maximum haematoma density was 86.9 ± 7.2 HU (69–100). Comparison of each measurement is shown in Table 3 for patients with and without haematoma expansion. Area under the receiver-operating-characteristic curve values for mean haematoma density was 0.54 (95% CI 0.48–0.65) HU and HU variability was 0.57 (95% CI 48–0.64). There was no significant correlation between the qualitative marker of heterogenous density and the HU variability (Spearman’s rho = −0.11, *p* = 0.16), but there was with HU coefficient of variation (Spearman’s rho = −0.20, *p* = 0.006).

**Table 3.** Haematoma Hounsfield units for in-hospital patients (*n* = 174).

Characteristic	24-h haematoma expansion (*n* = 76)	No 24-h haematoma expansion (*n* = 98)	*p*-Value
Mean Hounsfield units, mean (SD)	59.5 (3.8)	60.1 (5.0)	0.39
Hounsfield unit variability, mean (SD)	9.5 (1.4)	9.2 (1.1)	0.08
Maximum Hounsfield units, median (IQR)	88 (83–94)	86.5 (81–91)	0.06

Baseline haematoma volumes were significantly higher in patients with the presence of any CT marker than those without (Table 4). A number of CT markers were associated with 24-h haematoma expansion (Table 5) and 90-day poor functional outcome (mRS 3–6; Table 6) in univariable logistic regression analysis. After Bonferroni correction for multiple comparisons (adjusted significance threshold *p* < 0.00625), only the blend sign and the spot sign remained significantly associated with 24-h haematoma expansion, and heterogenous density, swirl sign, hypodensity and island sign remained significantly associated with poor 90-day functional outcome. On multivariable analysis (Tables 5 and 6), adjusting for age and baseline haematoma volume, the blend sign remained significantly associated with both 24-h haematoma expansion and poor 90-day functional outcome, whilst the island sign remained significantly associated with poor 90-day outcomes.

**Table 4.** baseline haematoma volume grouped by presence or absence of a CT marker.

CT marker		Baseline haematoma volume (ml)	IQR (ml)	*p*-Value
Heterogenous density	Present	21.2	12.1–34.5	<0.001
	Absent	7.3	3.9–13.5	
Hypodensities/swirl sign	Present	14.8	7.9–26.1	<0.001
	Absent	5.4	2.3–7.8	
Black hole sign	Present	17.3	11.3–35.0	<0.001
	Absent	7.8	3.9–15.9	
Blend sign	Present	15.9	10.0–28.2	0.03
	Absent	10.5	5.5–21.6	
Irregular shape	Present	18.6	10.5–34.2	<0.001
	Absent	6.1	3.5–11.8	
Island sign	Present	24.8	15.1–40.9	<0.001
	Absent	7.8	4.1–13.2	
Satellite sign	Present	21.1	10.5–31.2	<0.001
	Absent	7.9	4.2–15.5	
Spot sign	Present	15.1	8.2–27.4	<0.001
	Absent	7.7	5.5–24.3	

**Table 5.** Logistic regression analysis for association with 24-h haematoma expansion.

Outcome	Unadjusted OR (95%CI)	Unadjusted OR *p*-value	Adjusted OR (95%CI)^a^	Adjusted OR *p*-value
Age	1.03 (1.01–1.04)	<0.01	-	-
Antiplatelet use	1.69 (0.98–2.92)	0.06	2.25 (1.07–4.75)	0.03
Baseline haematoma volume	1.03 (1.01–1.04)	<0.01	-	-
Tranexamic acid	0.88 (0.53–1.46)	0.62	1.05 (0.65–1.70)	0.84
Heterogenous density	1.68 (1.00–2.83)	0.05	1.15 (0.67–1.99)	0.61
Swirl sign	1.86 (1.02–3.41)	0.04	1.05 (0.57–1.96)	0.87
Hypodensity	1.86 (1.02–3.41)	0.04	1.05 (0.57–1.96)	0.87
Black hole sign	1.21 (0.69–1.83)	0.64	0.75 (0.43–1.31)	0.32
Blend sign	12.49 (2.81–55.65)	<0.01	4.70 (1.79–12.38)	<0.01
Mean hounsfield units	0.97 (0.91–1.04)	0.37	0.95 (0.89–1.03)	0.21
Hounsfield unit variability	0.81 (0.64–1.03)	0.08	0.83 (0.64–1.06)	0.13
Irregular shape	1.89 (1.13–3.17)	0.02	1.34 (0.75–2.39)	0.32
Island sign	1.79 (1.04–3.10)	0.04	1.10 (0.59–2.05)	0.76
Satellite sign	1.52 (0.91–2.55)	0.11	1.12 (0.65–1.93)	0.67
Spot sign	2.46 (1.40–4.32)	<0.01	1.47 (0.87–2.50)	0.15

^a^Adjusted for age and baseline haematoma volume.

**Table 6.** Logistic regression analysis for association with poor functional outcome (mRS 3–6).

Outcome	Unadjusted OR (95% CI)	Unadjusted OR *p*-value	Adjusted OR (95%CI)^a^	Adjusted OR *p*-value
Age	1.04 (1.02–1.07)	<0.01	-	-
Antiplatelet use	2.25 (1.07–4.75)	0.03	1.19 (0.58–2.42)	0.64
Baseline haematoma volume	1.07 (1.04–1.10)	<0.01	-	-
Tranexamic acid	0.80 (0.46–1.40)	0.44	0.97 (0.56–1.69)	0.92
Heterogenous density	3.41 (1.77–6.57)	<0.01	1.94 (0.96–3.89)	0.06
Swirl sign	3.25 (1.77–5.96)	<0.01	1.94 (0.96–3.89)	0.06
Hypodensity	3.25 (1.77–5.96)	<0.01	1.48 (0.76–2.73)	0.27
Black hole sign	1.82 (0.99–3.32)	0.06	0.93 (0.49–1.79)	0.84
Blend sign	3.40 (0.76–15.19)	0.11	4.70 (1.79–12.38)	<0.01
Mean Hounsfield units	1.05 (0.97–1.13)	0.23	1.02 (0.94–1.12)	0.61
Hounsfield unit variability	1.17 (0.90–1.52)	0.24	1.35 (0.99–1.83)	0.06
Irregular shape	4.83 (2.56–9.1)	<0.01	2.91 (1.48–5.72)	<0.01
Island sign	4.88 (2.20–10.82)	<0.01	1.63 (0.75–3.54)	0.22
Satellite sign	2.30 (1.25–4.24)	<0.01	1.39 (0.73–2.65)	0.32
Spot sign	2.05 (1.08–3.88)	<0.01	0.99 (0.54–1.81)	0.96

^a^Adjusted for age and baseline haematoma volume.

**Figure 2.**
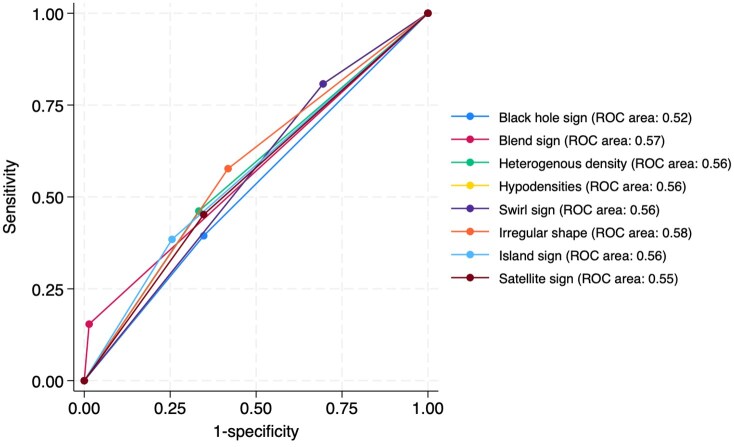
receiver operating characteristic curves for performance of each non-contrast CT marker to classify patients with haematoma expansion.

Supplemental Table S2 shows discriminative performance for each CT marker for haematoma expansion. Though infrequently observed (7.3%), the blend sign, when present, had a high positive predictive value. Area under the receiver-operating-characteristic curve ranged from 0.52 (0.46–0.58) for the black hole sign to 0.61 (0.54–0.67) for the spot sign but there were no significant differences (Chi squared test statistic χ^2^(6) = 3.02 with a corresponding *p*-value of 0.81; Figure 2).

The proportion of patients with and without haematoma expansion who received tranexamic acid was similar (*p* = 0.98), and tranexamic acid did not significantly reduce the risk of haematoma expansion (OR 0.88 [95% CI 0.53–1.46], *p* = 0.62), in keeping with the original trials. However, when stratified for the presence of each CT marker (Table 2), a significant interaction was observed between treatment with tranexamic acid and the presence of a spot sign on the likelihood of haematoma expansion (*p*_interaction_ = 0.01).

## Discussion

In this pooled descriptive analysis of patients from the STOP-AUST and STOP-MSU trials with baseline imaging within 2 h from symptom onset, we found that CT markers of haematoma expansion were frequently present in the ultra-early timeframe. Of the NCCT markers, we found a similar frequency of shape markers compared with the published literature.^10^ However, the density markers were more common in our patient population compared with previous reports. This may be due to the ultra-early timeframe and the likelihood that active bleeding and haematoma expansion is occurring at the time of imaging.^25^ Hypoattenuation on CT is considered an early finding due to lower concentration of haemoglobin in these regions.^26^ Subsequently, platelet aggregation and fibrin formation result in a seal, and eventual clot retraction, with increased density of haematocrit resulting in higher CT attenuation.^27^

We examined the association between CT imaging markers and haematoma expansion at 24-h, as well as poor functional outcome. Our primary analyses focused on unadjusted logistic regression models, in keeping with a descriptive epidemiological approach.^20^ However, given the strong and well-established prognostic role of age and baseline haematoma volume in both haematoma expansion and outcome, we also performed adjusted analyses for these two covariates, as performed in previous studies.^21–24^ Prior to performing this adjustment within our cohort, we assessed baseline haematoma volumes for patients with and without each marker and found significantly higher haematoma volumes in patients with CT markers. This suggests a potential confounding relationship, as CT markers may themselves be indicative of larger baseline haematoma volumes. This observation implies that these CT markers are not only associated with haematoma expansion at 24 h but may also reflect processes contributing to larger initial haematoma sizes. Accordingly, adjustment for baseline haematoma volume must be interpreted with caution, as it may violate the positivity assumption. If CT markers are themselves influenced by or strongly associated with baseline volume, adjusting for volume could introduce bias by controlling for a variable that lies on the causal pathway between the imaging marker and expansion.

The adjusted analyses demonstrate that several markers lose statistical significance for association with haematoma expansion and poor outcome after adjustment for age and baseline volume. These findings suggest that the prognostic utility of some CT markers may be partially explained by their association with larger baseline haematomas, rather than an independent predictive effect on expansion. We do not interpret these adjusted analyses as definitive estimates of causal effect but rather as sensitivity analyses that provide context for our unadjusted findings. They illustrate the complexity of interpreting associations between imaging features and clinical outcomes in intracerebral haemorrhage and underscore the need for caution when adjusting for variables such as baseline haematoma volume that may serve both as confounders and mediators.

We explored various measures of haematoma Hounsfield units as quantitative markers of the distribution of haematoma density.^28^ The association of haematoma Hounsfield units with haematoma expansion has been described in several other small studies, with inclusion of patients from 6 to 24 h from onset of symptoms.^12,29–31^ Our findings are similar to these studies, where haematoma expansion was associated with lower mean haematoma Hounsfield units. We found that the Hounsfield unit coefficient of variation was correlated with the qualitative marker of heterogenous density. However, neither marker had good diagnostic performance for predicting haematoma expansion. Further validation of this approach as a quantitative marker of haematoma density is required.

The ultra-early time period used in our study is particularly important, given that the majority of haematoma expansion has been shown to occur in the ultra-early timeframe.^21,32^ The neutral results of previous haemostatic or antifibrinolytic trials which include patients with particular CT markers of haematoma expansion, such as STOP-AUST, SPOTLIGHT (factor VIIa in spot sign positive patients <6.5 h) and TRAIGE (tranexamic acid in spot sign, blend sign or black hole sign positive patients <6 h) may be related to the inclusion of patients with these signs in the later timeframe.

The STOP-AUST and STOP-MSU trials both showed neutral effect of tranexamic acid on 24-h haematoma expansion. In the present cohort, the spot sign was the only marker associated with benefit of tranexamic acid in reducing haematoma growth. This supports a recent individual patient data meta-analysis which showed effect on the reduction of haematoma expansion in spot sign positive patients, up to 4.5 h from onset, but particularly in the sub-2 h group (aRR 0.51 [95% CI 0.32–0.79]).^33^ The meta-analysis did include patients from STOP-AUST (as in the present study), but we now included an additional 152 patients from STOP-MSU with spot sign data. These results require further exploration given the individual trials were neutral but support the utility of the spot sign for patients presenting in the ultra-early timeframe.^3,5,9^

Strengths of this study include inclusion of patients recruited within the ultra-early timeframe, 2 h from symptom onset, from emergency department presentations and mobile stroke units, allowing generalisability to both pre-hospital and in-hospital patients. Second, the use of a dataset from two international trials increases the geographic and ethnic generalisability of our study. Another strength is the utilisation of a central imaging lab for imaging analysis, with all volumes measured by validated semi-automated techniques. The timing of the 24-h scans were prospectively dictated by trial protocol, ensuring a more consistent dataset than routine observational series would enable.

Limitations of this study include its small sample size and it being a secondary analysis of two randomised controlled trials. Given this, and the descriptive nature of the study, regression analyses could not be adjusted for confounders. These findings cannot be extrapolated to infratentorial haemorrhage (*n* = 1 in our population) or patients taking anticoagulants (exclusion criteria). A key limitation is the frequent co-occurrence of CT markers within individual patients, which limits the ability to isolate the unique discriminative value of each marker. Of the 246 patients included, 44 patients were enrolled through a mobile stroke unit. While mobile stroke units allow for ultra-early diagnosis of patients with haemorrhagic (and ischaemic) stroke, the quality of the CT imaging obtained for these patients is often inferior to a standard hospital CT image and this may have impacted our results. In the assessment of haematoma Hounsfield units, the semi-automated software has a threshold limit of 40–100 Hounsfield units for intracranial haemorrhage. Therefore, values outside of this range are excluded in segmentation of the haematoma.

In conclusion, CT markers are commonly present in the ultra-early period of intracerebral haemorrhage. Our analysis showed that the spot-sign and blend sign were associated with subsequent haematoma expansion. However, there were variable associations with subsequent functional outcome and the discriminative utility of these markers alone in the early timeframe is generally insufficient. Larger pooled studies may be required to further assess diagnostic and prognostic performance of these markers. We observed that patients with a spot sign had increased rates of haematoma expansion, and a potential benefit of tranexamic acid. While we are unable to a draw causal inference, our findings support the rationale for further trials incorporating this prognostic marker of active bleeding.

## Supplementary Material

sj-docx-1-eso-23969873251355938

## Data Availability

Deidentified data will be made available on reasonable request by email communication to the corresponding author following review and approval of a research proposal by the trial executive committee, with a signed data access agreement.
