# Supplementary material for: Ultra-early computed tomography markers of haematoma expansion: Potential trial targets?
Source: Eur Stroke J. 2026 Jan 1;11(1):23969873251355938. doi: 10.1093/esj/23969873251355938 (PMC12866209; doi:10.1093/esj/23969873251355938)
Supplement: sj-docx-1-eso-23969873251355938 [file sj-docx-1-eso-23969873251355938.docx]

## Supplementary material

## STOP-AUST eligibility criteria

Inclusion criteria:

- Patients presenting with an acute ICH
- Contrast extravasation within the haemorrhage, ‘spot sign’, evaluated from the CTA according to three criteria, all of which must be present:
  - Serpiginous or spot-like appearance within the margin of a parenchymal haematoma without connection to an outside vessel;
  - The density (in Hounsfield units) should be greater than that of the background haematoma (site investigators are not required to document the density); and
  - No hyperdensity at the corresponding location on noncontrast CT.
- Age ≥18 years
- Treatment can commence within one hour of initial CT and within 4.5 hours of symptom onset (or in patients with unknown time of symptom onset, the time patient was last known to be well)
- Informed consent has been received in accordance to local ethics committee requirements

Exclusion criteria:

- Glasgow coma scale (GCS) total score of <8
- Brainstem ICH
- ICH volume >70 ml as measured by the ABC/2 method
- ICH known or suspected by study investigator to be secondary to trauma, aneurysm, vascular malformation, haemorrhagic transformation of ischemic stroke, cerebral venous thrombosis, thrombolytic therapy, tumour, or infection
- Contrast already administered within 24 hours prior to initial CT or contraindication to imaging with CT contrast agents (e.g. known or suspected iodine allergy or significant renal failure)
- Any history or current evidence suggestive of venous or arterial thrombotic events within the previous 12 months, including clinical, ECG, laboratory, or imaging findings. Clinically silent chance findings of old ischemia are not considered exclusion.
- Hereditary or acquired haemorrhagic diathesis or coagulation factor deficiency
- Use of heparin, low-molecular weight heparin, GPIIb/IIIa antagonist, or oral anticoagulation (e.g. warfarin, factor Xa inhibitor, thrombin inhibitor) within the previous 14 days, irrespective of laboratory values
- Pregnancy (women of childbearing potential must be tested)
- Planned surgery for ICH within 24 hours
- Concurrent or planned treatment with haemostatic agents (e.g. prothrombin complex concentrate, vitamin K, fresh frozen plasma, or platelet transfusion)
- Participation in any investigational study in the last 30 days
- Known terminal illness or planned withdrawal of care or comfort care measures
- Any condition that could, in the judgment of the investigator, impose hazards to the patient if study therapy is initiated, or affect the participation of the patient in the study.

## STOP-MSU eligibility criteria

Inclusion criteria:

- Patients presenting with an acute ICH
- Age ≥18 years
- Treatment can commence within 2 hours of symptom onset (or in patients with unknown time of symptom onset, the time patient was last known to be well)
- Consent can be obtained from the participant or person responsible

Exclusion criteria:

- GCS total score <8
- Brainstem ICH
- ICH volume >70ml as measured by the ABC/2 method
- ICH known or suspected to be secondary to trauma, aneurysm, vascular malformation, haemorrhagic transformation of ischaemic stroke, cerebral venous thrombosis, thrombolytic therapy, tumour or infection
- Any history or current evidence suggestive of venous or arterial thrombotic events within the previous 90 days. Silence chance old ischaemia is not considered an exclusion
- Hereditary or acquired haemorrhagic diathesis or coagulation factor deficiency. Use of heparin, low-molecular weight heparin, GPIIb/IIIa antagonist, or oral anticoagulation (e.g. warfarin/vitamin K antagonists, factor Xa inhibitor, thrombin inhibitor) within the previous 72 hours. Warfarin/vitamin K antagonists must also have an INR of <1.3 prior to randomisation.
- Pregnancy (women of childbearing potential must be tested)
- Planned surgery for ICH within 24 hours
- Concurrent or planned treatment with haemostatic agents (e.g. prothrombin complex concentrate, vitamin K, fresh frozen plasma, or platelet transfusion)
- Participation in any investigational study in the last 30 days
- Known terminal illness or planned withdrawal of care or comfort care measures.
- Any condition that, in the judgment of the investigator could impose hazards to the patient if study therapy is initiated or affect the participation of the patient in the study.

### Table S1: Interrater reliability for CT markers

| **CT marker** | **Weighted kappa** |
| --- | --- |
| Heterogeneous density | 0.87 |
| Swirl sign | 0.86 |
| Hypodensity | 0.82 |
| Black hole sign | 0.86 |
| Blend sign | 0.87 |
| Fluid level | 1.00 |
| Irregular shape | 0.87 |
| Island sign | 0.90 |
| Satellite sign | 0.87 |
| Spot sign | 0.87 |

### Table S2: Diagnostic performance of CT markers for 24-hour haematoma expansion

|  | **Sensitivity (95%CI)** | **Specificity (95%CI)** | **Positive predictive value (95%CI)** | **Negative predictive value (95%CI)** | **ROC area (95%CI)** |
| --- | --- | --- | --- | --- | --- |
| Heterogenous density | 0.46 (0.36-0.56) | 0.67 (0.58-0.74) | 0.50 (0.43-0.58) | 0.62 (0.57-0.67) | 0.56 (0.50-0.63) |
| Hypodensities/swirl sign | 0.81 (0.72-0.88) | 0.31 (0.23-0.39) | 0.46 (0.43-0.50) | 0.68 (0.57-0.77) | 0.56 (0.50-0.63) |
| Black hole sign | 0.39 (0.30-0.49) | 0.65 (0.58-0.3) | 0.46 (0.38-0.54) | 0.59 (0.54-0.64) | 0.52 (0.46-0.58) |
| Blend sign | 0.15 (0.09-0.24) | 0.99 (0.95-1.00) | 0.89 (0.65-0.97) | 0.61 (0.59-0.64) | 0.57 (0.53-0.63) |
| Irregular shape | 0.58 (0.48-0.67) | 0.58 (0.50-0.66) | 0.51 (0.44-0.57) | 0.65 (0.59-0.71) | 0.58 (0.52-0.64) |
| Island sign | 0.38 (0.29-0.48) | 0.74 (0.66-0.81) | 0.53 (0.43-0.62) | 0.62 (0.57-0.66) | 0.56 (0.51-0.62) |
| Satellite sign | 0.45 (0.35-0.55) | 0.65 (0.57-0.73) | 0.49 (0.41-0.57) | 0.61 (0.56-0.66) | 0.55 (0.49-0.61) |
| Spot sign | 0.57 (0.46-0.68) | 0.65 (0.56-0.73) | 0.56 (0.49-0.63) | 0.66 (0.60-0.72) | 0.61 (0.54-0.67) |

### Figure S1: Flow diagram for patient inclusion


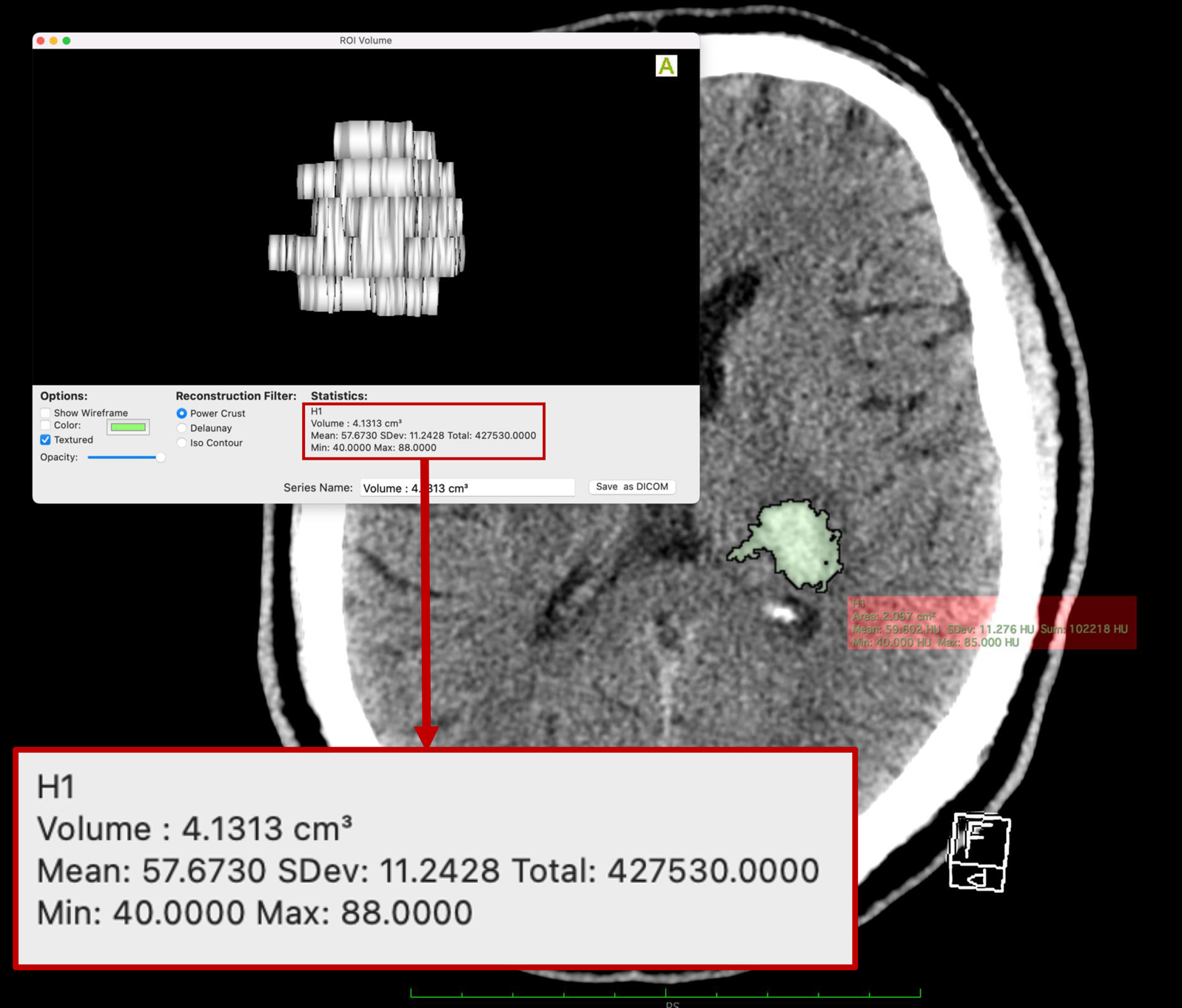


### Figure S2: Example of segmentation and Hounsfield unit output from semi-automated program
